# Reduced FBXW7 expression in pancreatic cancer correlates with poor prognosis and chemotherapeutic resistance via accumulation of MCL1

**DOI:** 10.18632/oncotarget.22634

**Published:** 2017-11-06

**Authors:** Norihiro Ishii, Kenichiro Araki, Takehiko Yokobori, Dorgormaa Gantumur, Takahiro Yamanaka, Bolag Altan, Mariko Tsukagoshi, Takamichi Igarashi, Akira Watanabe, Norio Kubo, Yasuo Hosouchi, Hiroyuki Kuwano, Ken Shirabe

**Affiliations:** ^1^ Department of Hepatobiliary and Pancreatic Surgery, Gunma University, Graduate School of Medicine, Showamachi, Maebashi, Japan; ^2^ Department of General Surgical Science, Gunma University, Graduate School of Medicine, Showamachi, Maebashi, Japan; ^3^ Research Program for Omics-Based Medical Science, Division of Integrated Oncology Research, Gunma University Initiative for Advanced Research, Maebashi, Japan; ^4^ Department of Oncology Clinical Development, Gunma University, Graduate School of Medicine, Maebashi, Japan; ^5^ Department of Surgery and Laparoscopic Surgery, Gunma Prefecture Saiseikai-Maebashi Hospital, Maebashi, Japan

**Keywords:** chemosensitivity, ubiquitin ligase, tumor suppressor, MCL1, nab-paclitaxel

## Abstract

Pancreatic cancer is a highly malignant tumor type with poor outcomes, and elucidation of the mechanisms involved in cancer progression and therapeutic resistance is critical. FBXW7 is a key regulator of tumor malignant potential, and its substrate MCL1 regulates therapeutic resistance in human malignancies. Therefore, determination of the relevance of FBXW7 expression is critical for improving patient outcomes. In this study, we investigated the function and clinical significance of FBXW7 in pancreatic cancer. FBXW7 expression was evaluated by immunohistochemistry in 122 pancreatic cancer tissues. Reduced FBXW7 expression was significantly associated with advanced venous invasion, high MCL1 expression, enhanced Ki-67 expression, and poor prognosis and was an independent poor prognostic factor. Among patients who underwent gemcitabine treatment after surgery, reduced FBXW7 expression was also significantly associated with poor prognosis. Knockdown of FBXW7 *in vitro* enhanced cell proliferation, and migration, and invasion abilities and promoted gemcitabine and nab-paclitaxel chemoresistance in pancreatic cancer cells. Moreover, FBXW7-knockdown cells showed accumulation of MCL1, and the enhanced chemoresistance observed in FBXW7-knockdown cells was eliminated by MCL1 suppression. These results suggested that FBXW7 was associated with cancer progression and mediated sensitivity to gemcitabine and nab-paclitaxel via MCL1 accumulation in pancreatic cancer. Thus, the FBXW7/MCL1 axis may be a promising therapeutic tool to overcome refractory pancreatic cancer.

## INTRODUCTION

Pancreatic cancer is a highly malignant tumor type with poor outcomes; the 5-year overall survival rate, including unresectable and metastatic cancer, is less than 10% [1, 2]. Although multidisciplinary treatment, including new chemotherapy, such as gemcitabine plus nab-paclitaxel [3] and FOLFIRINOX [4], is generally performed, the incidence of pancreatic cancer remains nearly equal to its mortality rate. Pancreatic cancer is characterized by rapid progression, early metastasis, and limited response to chemotherapy and radiotherapy [5]. Therefore, to improve the prognosis of patients with pancreatic cancer, it is important to elucidate the mechanisms underlying therapeutic resistance and develop new therapeutic targets.

F-box and WD repeat domain-containing 7 (FBXW7), which is a substrate recognition subunit of the Skp1-Cul1-F box ubiquitin ligase complex, is a key regulator of proliferation, invasion, apoptosis, and chemotherapeutic resistance in human malignancies and functions via the degradation of oncoproteins, including c-Myc and myeloid leukemia cell differentiation protein (MCL1), in a proteasome-dependent manner [6, 7]. Thus, FBXW7 is considered an important tumor suppressor in various cancers [8]. In clinical samples, reduced expression of FBXW7 has been reported to be associated with poor prognosis and cancer progression in various human solid cancers, such as gastric cancer [9], esophageal cancer [10], colorectal cancer [11], and cholangiocarcinoma [12, 13], due to the accumulation of several oncoproteins. In pancreatic cancer, a recent report showed that Ras/Raf/MEK/ERK activation induced by KRAS mutations, which are frequently observed in pancreatic cancer, plays important roles in reducing FBXW7 expression [14]. ERK directly phosphorylates FBXW7, which leads to the self-ubiquitination and proteosomal degradation of FBXW7 itself. This low FBXW7 protein expression results in accumulation of multiple FBXW7 substrates in pancreatic cancer. Moreover, although the mechanisms regulating FBXW7 expression are gradually being elucidated, studies on the impact of FBXW7 expression and its substrate in pancreatic cancer are limited.

MCL1, which is a target of FBXW7 and a member of the BCL2 protein family, is an anti-apoptotic protein that has been reported to be overexpressed in pancreatic cancer cells and clinical tissue samples and is correlated with advanced disease [15]. Furthermore, recent studies have suggested that MCL1 regulates not only apoptotic cell death but also responses to certain chemotherapeutic agents, particularly antitubulin chemotherapy [16, 17]. Therefore, it is important to investigate the role of FBXW7 expression, which regulates MCL1, in pancreatic cancer.

Accordingly, the aim of this study was to identify the function and clinical significance of FBXW7 in pancreatic cancer. In the present study, we examined FBXW7 expression in 122 pancreatic cancer tissues using immunohistochemistry to clarify the clinical significance of FBXW7 expression. Moreover, the effects of FBXW7 downregulation by RNA interference (RNAi) were examined in pancreatic cancer cells to evaluate whether FBXW7 played important roles in proliferation, migration, invasion, and chemoresistance. Furthermore, we demonstrated MCL1 is a key substrate of FBXW7 involved in chemotherapeutic resistance in pancreatic cancer.

## RESULTS

### Immunohistochemical analysis of FBXW7 expression in patients with pancreatic cancer

**Figure 1:**
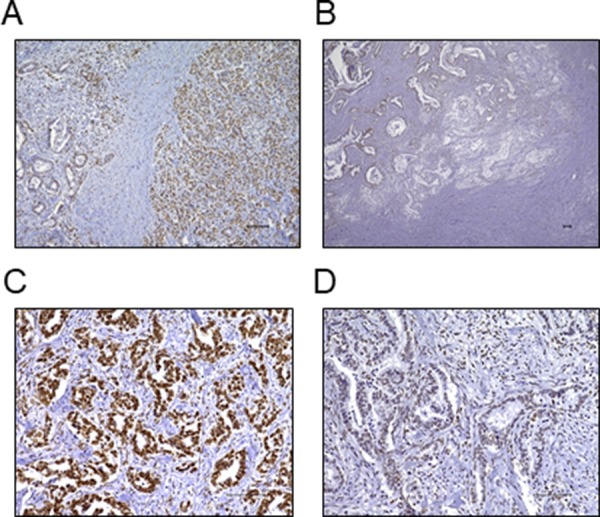
Immunohistochemical staining of FBXW7 in pancreatic cancer tissue (**A**) FBXW7 expression was observed in the nuclei of cells. FBXW7 expression in cancer tissue tended to be lower than that in adjacent noncancerous tissue (original magnification, 100×). (**B**) FBXW7 expression at the cancer invasion front was lower than that in the primary tumor (original magnification, 40×). (**C**) A representative sample of high FBXW7 expression (original magnification, 200×). (**D**) A representative sample of low FBXW7 expression (original magnification, 200×). All scar bars are 100 μm.

FBXW7 expression was immunohistochemically evaluated in 122 pancreatic cancer tissues and was found to be mainly expressed in the nucleus. Hence, we performed nuclear staining of cancer cells as for determining FBXW7 expression. Nuclear FBXW7 expression in cancer tissues tended to be lower than that in adjacent noncancerous tissues (Figure 1A). Additionally, FBXW7 expression at the cancer invasion front was lower than that in the center of the primary tumor (Figure 1B). In this study, 68 samples were included in the high FBXW7 expression group (Figure 1C), and 54 samples were included in the low FBXW7 expression group (Figure 1D).

### Clinical significance of nuclear FBXW7 expression in patients with pancreatic cancer

The associations between clinicopathological characteristics and FBXW7 expression are presented in Table 1. Low FBXW7 expression was significantly associated with higher venous invasion (*P* = 0.037), and a similar trend was observed in lymphatic invasion, although the difference was not significant (*P* = 0.081). Furthermore, the expression levels of FBXW7 and MCL1 were significantly inversely correlated (*P* = 0.032). Thus, Low FBXW7 expression was associated with high MCL1 expression. Representative relationships between FBXW7 and MCL1 expression are shown in Supplementary Figure 1. Samples with low FBXW7 expression showed enhanced MCL1 expression (Supplementary Figure 1A), whereas samples with high FBXW7 expression showed decreased MCL1 expression (Supplementary Figure 1B).

**Table 1:** Clinicopathological characteristics according to FBXW7 expression in 122 pancreatic cancer samples

Factors	FBXW7 expression		*p*-value
	High (*n* = 68)	Low (*n* = 54)	
Age, median (range)	69 (36–87)	69 (36–82)	0.299
Sex			0.166
Male	33	33	
Female	35	21	
Histological type			0.521
Well	10	6	
Moderately, Poorly	49	42	
T factor (UICC)			0.343
T1, 2	7	3	
T3, 4	61	51	
Tumor size			0.907
≤ 40 mm	51	40	
> 40 mm	17	14	
Lymph node metastasis			0.432
Absent	18	11	
Present	50	43	
Venous invasion			0.037^*^
v0, 1	35	18	
v2, 3	32	36	
Lymphatic invasion			0.081
ly0, 1	38	22	
ly2, 3	29	32	
Perineural invasion			0.377
ne0, 1	21	13	
ne2, 3	46	41	
TNM stage (UICC)			0.546
I, IIA	17	11	
IIB, III, IV	51	43	
Reccurence			0.510
Absent	20	13	
Present	48	41	
MCL1 expression			0.032^*^
Low	47	27	
High	21	27	
Ki-67 index, median (range)	38 (0-134)	62 (3-344)	0.026^*^

Abbreviations: UICC, Union for International Cancer Center.

^*^p < 0.05.

Next, Ki-67 expression was evaluated to validate the relationships between FBXW7 expression and proliferation potential. The proportion of Ki-67-positive cells as a proliferation marker was significantly higher in the low FBXW7 expression group than in the high FBXW7 expression group (*P* = 0.026). Additionally, localization of nuclear FBXW7 expression and Ki-67 expression was inversely correlated, as shown by evaluating sequential sections of tissue samples by immunohistochemistry (Supplementary Figure 2).

**Figure 2:**
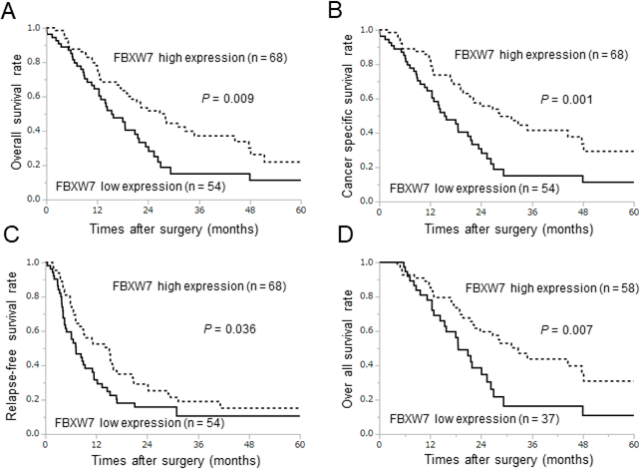
Survival curves according to FBXW7 expression in pancreatic cancer All survival curves were obtained using the Kaplan-Meier methods. (**A**, **B**) Overall survival rates and cancer-specific survival rates. (**C**) Relapse-free survival rates. (**D**) Prognosis in relation to gemcitabine treatment after surgery or recurrence in the low and high FBXW7 expression groups.

The association between prognosis and FBXW7 expression was evaluated by the Kaplan-Meier method. The overall survival and cancer-specific survival rates were significantly lower in patients with low FBXW7 expression than in patients with high FBXW7 expression (*P* = 0.009 and *P* = 0.001, respectively; Figure 2A, B). Similarly, the relapse-free survival rate was also significantly lower in patients with low FBXW7 expression (P = 0.036; Figure 2C). Furthermore, we evaluated the influence of chemotherapy with gemcitabine, a major drug for pancreatic cancer chemotherapy, on prognosis according to FBXW7 expression. A total of 95 patients received chemotherapy as an adjuvant chemotherapy or to treat recurrence, and 27 patients did not receive chemotherapy. In patients who underwent gemcitabine treatment, the overall survival rate in the low FBXW7 expression group was significantly shorter than that in the high expression group (*P* = 0.007; Figure 2D). These data suggested that low nuclear expression of FBXW7 in cancer tissue may be related to chemoresistance.

Next, we evaluated the significance of FBXW7 expression as a prognostic factor using the Cox proportional hazards model. Univariate and multivariate analyses for overall survival are show in Table 2. Univariate analysis showed that low FBXW7 expression was a poor prognostic factor (*P* = 0.011), as were histological type (*P* = 0.031), T factor (*P* = 0.001), and venous invasion (*P* < 0.0001). Multivariate analysis showed that low FBXW7 expression was an independent prognostic factor of poor survival (hazard ratio [HR] = 1.72; 95% confidence interval [CI] = 1.06–2.79; *P* = 0.029), similar to existing clinicopathological factors, such as venous invasion (*P* = 0.003). Similarly, univariate and multivariate analyses for relapse-free survival also showed that low FBXW7 expression was an independent prognostic factor (HR = 1.55; 95% CI = 1.01–2.37; *P* = 0.047, Supplementary Table 1).

**Table 2:** Univariate and multivariate analyses of variables related to overall survival, as determined using Cox proportional hazards models

Variables	Univariate analysis			Multivariate analysis		
	HR	95% CI	*p* value	HR	95% CI	*p* value
Age (< 70 vs. ≥ 70)	1.02	0.66–1.57	0.899	−	−	−
Sex (Male vs. Female)	0.93	0.61–1.44	0.774	−	−	−
Histological type (Well vs. Moderately, Poorly)	2.09	1.06–4.76	0.031^*^	1.05	0.56−2.20	0.881
T factor (UICC) (T1, 2 vs. T3, 4)	3.89	1.61–12.82	0.001^*^	1.80	0.62−7.66	0.306
Lymph node metastasis (Absent vs. Present)	1.19	0.72–2.06	0.505	−	−	−
Venous invasion (v0,1 vs. v2,3)	2.53	1.61–4.07	< 0.0001^*^	2.52	1.50−4.40	0.003^*^
Lymphatic invasion (ly0,1 vs. ly2,3)	1.52	099–2.35	0.053	−	−	−
Perineural invasion (ne0,1 vs. ne2,3)	1.39	0.86–2.33	0.176	−	−	−
FBXW7 (High vs. Low)	1.78	1.14–2.79	0.011^*^	1.66	1.03−2.66	0.039^*^

Abbreviations: HR, hazard ratio; CI, confidence interval; UICC, Union for International Cancer Center.

^*^p < 0.05.

### RNAi targeting FBXW7 enhanced proliferation, migration, invasion, and chemoresistance and caused MCL1 accumulation in pancreatic cancer cells

**Figure 3:**
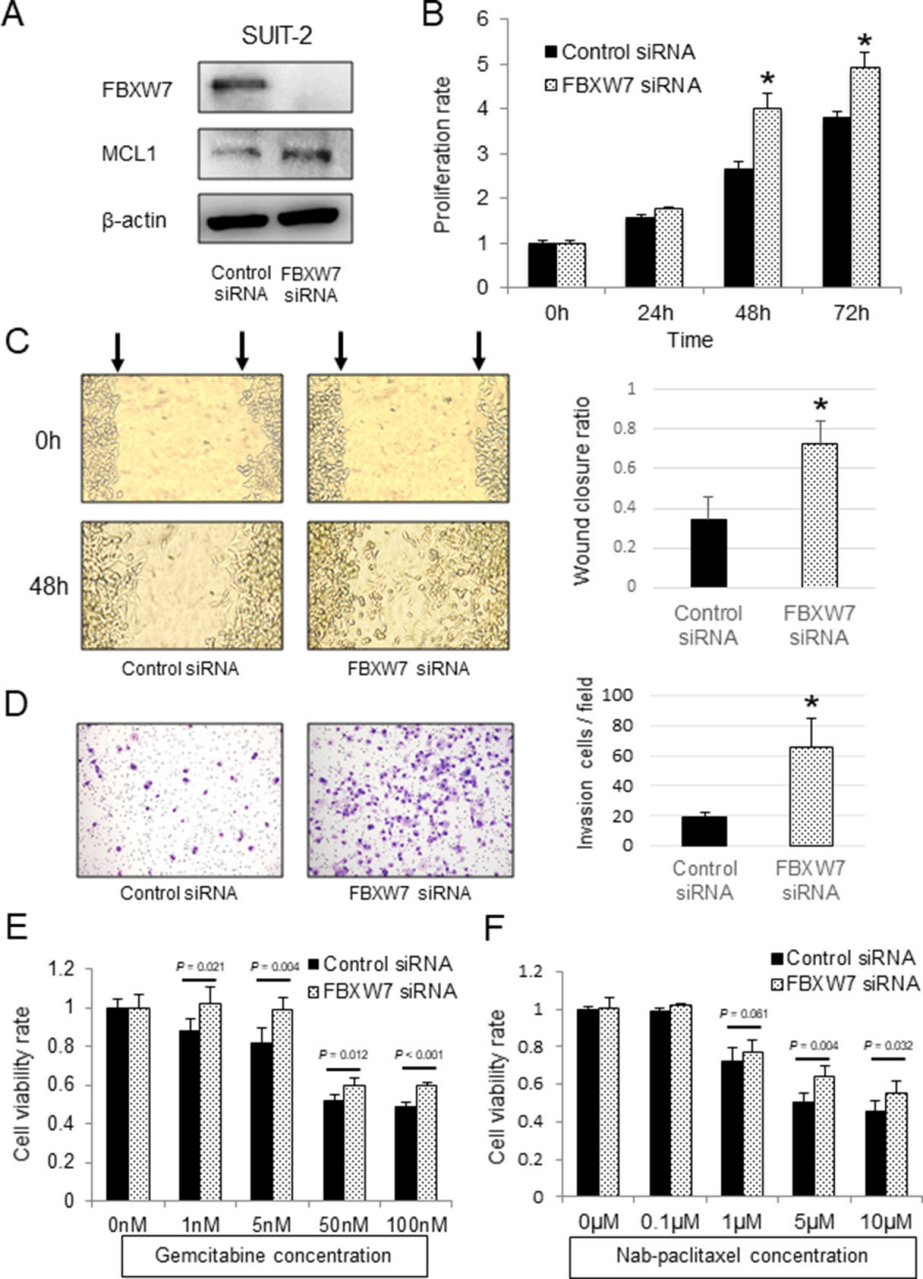
*In vitro* functional analysis of FBXW7 in SUIT-2 pancreatic cancer cells transfected with FBXW7-specific siRNA (**A**) FBXW7 and MCL1 expression levels in SUIT-2 cells transfected with FBXW7-specific siRNA were evaluated by western blotting. β-actin was used as the internal control. (**B**) Cell proliferation ability of SUIT-2 cells transfected with FBXW7-specific siRNA was analyzed using CCK-8 assays. (**C**) Cell migration ability of the FBXW7-specific siRNA group was evaluated by wound healing assays. (**D**) Cell invasion ability of the FBXW7-specific siRNA group was evaluated by Matrigel invasion assay. (**E**, **F**) Cell viabilities after 48 h of treatment with gemcitabine and nab-paclitaxel were evaluated using CCK-8 assays. FBXW7 knockdown cells revealed enhanced resistance to both gemcitabine and nab-paclitaxel compared with control cells. CCK-8, Cell Counting Kit-8; ^*^P < 0.05.

FBXW7 protein was expressed in SUIT-2 pancreatic cancer cells. We then knocked down FBXW7 expression in SUIT-2 cells by transfection with FBXW7-specific siRNA. FBXW7 suppression was confirmed by western blotting (Figure 3A). The proliferation ability of FBXW7-knockdown cells was significantly enhanced in comparison with that in cells transfected with control siRNA (Figure 3B). Additionally, FBXW7-knockdown cells showed enhanced cell migration and invasion compared with control cells (Figure 3C, 3D). Analysis of the relationship between FBXW7 expression and chemotherapy with gemcitabine in clinical samples showed that FBXW7 expression may be related to chemosensitivity.

We next evaluated the effects of FBXW7 knockdown on resistance to gemcitabine and nab-paclitaxel, which are used in first-line chemotherapy regimens for patients with advanced pancreatic cancer. FBXW7-knockdown cells showed enhanced resistance to both gemcitabine and nab-paclitaxel compared with control cells (Figure 3E, 3F). Furthermore, the anti-apoptotic protein MCL1, which is a degradation substrate of FBXW7, was evaluated by western blotting. The results showed that MCL1 was accumulated in FBXW7-knockdown cells in comparison with control cells (Figure 3A).

### The enhanced gemcitabine and nab-paclitaxel resistances in FBXW7-knockdown cells were eliminated by MCL1 suppression

**Figure 4:**
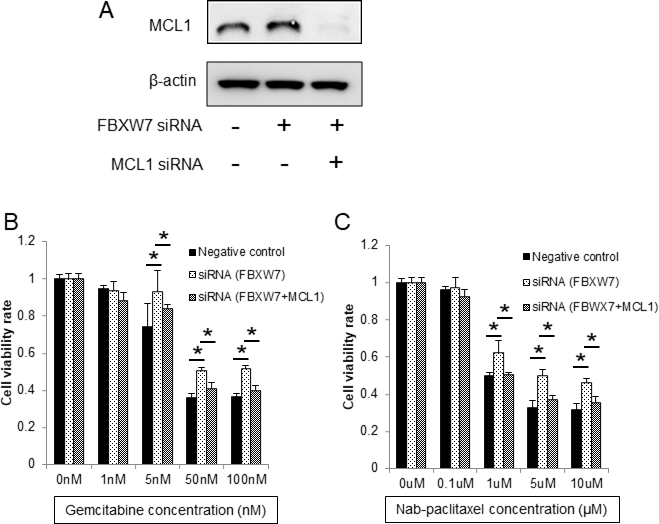
Enhanced chemoresistance in FBXW7-knockdown cells was eliminated by MCL1 suppression (**A**) MCL1 suppression was evaluated by western blotting. β-actin was used as the internal control. (**B**, **C**) Cell viabilities after 48 h of treatment with gemcitabine and nab-paclitaxel were evaluated using CCK-8 assays. Enhanced chemoresistance to gemcitabine and nab-paclitaxel in FBXW7-knockdown cells was eliminated by MCL1 suppression CCK-8, Cell Counting Kit-8; ^*^P < 0.05.

Next, to evaluate whether enhanced chemoresistance to gemcitabine and nab-paclitaxel in FBXW7-knockdown cells was dependent on MCL1 accumulation, we suppressed MCL1 expression using MCL1-specific siRNA in FBXW7-knockdown cells. MCL1 suppression was confirmed by western blotting (Figure 4A). Enhanced chemoresistance to gemcitabine and nab-paclitaxel in FBXW7-knockdown cells was eliminated by MCL1 suppression (Figure 4B, 4C).

## DISCUSSION

Here, we demonstrated that low FBXW7 expression in pancreatic cancer tissue was associated with cancer progression and was an independent factor predicting poor prognosis. Additionally, an inverse relationship between FBXW7 and MCL1 or Ki-67 expression was observed by immunohistochemistry. Moreover, among patients with gemcitabine treatment, those in the low FBXW7 expression group showed significantly shorter overall survival than those in the high FBXW7 expression group, suggesting that FBXW7 may mediate chemoresistance. In our *in vitro* analysis, we found that knockdown of FBXW7 enhanced cell proliferation, migration, and invasion abilities, and decreased sensitivity to gemcitabine and nab-paclitaxel in pancreatic cancer cells. Furthermore, the decreased chemosensitivity in FBXW7-knockdown cells was recovered by MCL1 suppression, suggesting that reduced FBXW7 levels enhanced chemoresistance via accumulation of the anti-apoptotic protein MCL1.

In previous studies, low expression of FBXW7 in cancer tissue was found to be associated with poor prognosis and cancer progression in several solid cancers, including gastric cancer [9], colorectal cancer [11], esophageal cancer [10, 18], non-small cell lung cancer [19], cholangiocarcinoma [12, 13], and hepatocellular carcinoma [20]. These results suggested that FBXW7 status in cancer tissue may be a potential prognostic marker. In our study, low FBXW7 expression was significantly associated with venous invasion and poor prognosis and was therefore an independent factor predicting poor prognosis. Thus, FBXW7 expression in pancreatic cancer may be a promising prognostic marker, consistent with previous reports in other cancers.

FBXW7 regulates various proteins, including c-Myc, cyclin E, mTOR, c-Jun, MCL1, and Notch, via the ubiquitin-proteasome system [6, 21]. In previous studies, these proteins were reported as oncoproteins whose overexpression is associated with poor prognosis, aggressive proliferation, migration, invasion, and metastatic potential in various cancers [22–24]. Moreover, a recent study showed that β-catenin, which regulates the Wnt signaling pathway and is related to pancreatic cancer malignancy, is a target of FBXW7 [25]. Thus, these data suggested that low expression and/or loss of function of FBXW7 leads to accumulation of various oncoproteins and activation of the Wnt signaling pathway via β-catenin accumulation, suggesting that FBXW7 regulates the malignant potential of cancers, including pancreatic cancer. In the present study, we demonstrated, for the first time, that the proliferative activity of cells in pancreatic cancer samples with low FBXW7 expression was enhanced and that the degree of venous invasion in cells with low FBXW7 expression was more advanced compared with that in cells with high FBXW7 expression. Furthermore, we validated that FBXW7 suppression using siRNA enhanced cells proliferation, migration, and invasion abilities *in vitro*. Recently, Jin and colleagues demonstrated that FBXW7 suppresses enhancer of zeste homolog 2 (EZH2) activity and inhibits tumor migration and invasion via degradation of EZH2 in pancreatic cancer cells [26]. Thus, since FBXW7 mediates diverse proteins that regulate malignant potential, such as tumor proliferation, migration, and invasion, which are important for pancreatic cancer progression, FBXW7 induction may be a novel therapeutic strategy to suppress pancreatic cancer development and progression.

As previously described, FBXW7 regulates the degradation of several oncoproteins related to the not only tumor aggressiveness but also therapeutic resistance [19, 27–29]. MCL1 is an important target substrate of FBXW7 and is associated with apoptosis [16]. In previous studies, elevated MCL1 levels were found to induce resistance to antitubulin therapies, such as paclitaxel and vincristine [17, 19], and MCL1 gene silencing induced apoptosis and increased chemosensitivity to gemcitabine in pancreatic cancer [30]. Recently, nab-paclitaxel, new antitubulin agent, has been added to some chemotherapy regimens for the treatment of pancreatic cancer [3, 31]. Additionally, gemcitabine has been used as a key drug for the treatment of pancreatic cancer for many years [32]. Thus, MCL1, which is associated with sensitivity to both antitubulin therapy and gemcitabine, is an important protein mediating chemoresistance in pancreatic cancer. In the present study, we found an inversed relationship between FBXW7 and MCL1 expression in clinical samples and showed that FBXW7 suppression decreased sensitivity to both gemcitabine and nab-paclitaxel and elevated MCL1 protein levels *in vitro*. Additionally, we showed that the decreased sensitivity to both agents induced by FBXW7 suppression was recovered by MCL1 suppression. These results revealed that MCL1 may be a key protein involved in chemoresistance induced by FBXW7 knockdown in pancreatic cancer cells. Furthermore, in pancreatic cancer clinical samples, among patients who received gemcitabine treatment as adjuvant therapy or after recurrence, the overall survival rate was significantly lower in the low FBXW7 expression group than in the high FBXW7 expression group. This suggested that the poor prognosis in patients with low FBXW7 expression may be caused by chemoresistance. Although we could not evaluate the relationship between FBXW7 expression and nab-paclitaxel efficacy because there was no nab-paclitaxel treatment group in this study, we expect that tumors will be refractory to nab-paclitaxel treatment in patients with low FBXW7 expression. Thus, FBXW7 expression status in cancer tissue may be a predictive marker of standard chemotherapy for pancreatic cancer. The present study is the first report demonstrating an association between FBXW7 expression and chemosensitivity in pancreatic cancer. Although chemotherapeutic resistance in pancreatic cancer is a serious problem and occurs through various molecular pathways [33], our data showed that FBXW7 and its substrate MCL1 are important factors involved in chemotherapeutic resistance and are expected to be candidate therapeutic targets for overcoming therapeutic resistance.

In conclusion, we found that FBXW7 expression levels in primary tumor tissues could predict prognosis in patients with pancreatic cancer. In addition, reduced FBXW7 expression enhanced tumor cell proliferation, migration, and invasion and the sensitivity of pancreatic cancer cells to gemcitabine and nab-paclitaxel was regulated by FBXW7 via MCL1 accumulation. These results suggested that evaluation of FBXW7 expression may be a promising prognostic marker and that the FBXW7/MCL1 axis may be employed as a therapeutic tool to overcome refractory pancreatic cancer.

## MATERIALS AND METHODS

### Clinical samples

Primary pancreatic cancer tissues were obtained from 122 patients with pancreatic cancer who underwent curative surgical resection at the Department of Gunma University Hospital (Maebashi, Japan) and Saiseikai Maebashi Hospital (Maebashi, Japan) between 1999 and 2012. There were 66 men and 56 women, and the median age was 67.4 years (range, 36–87 years). There were no patients who had received neoadjuvant chemotherapy or irradiation prior to surgical resection. Ninety-eight patients received adjuvant chemotherapy by gemcitabine, tegafur/gimeracil/oteracil (S-1), or tegafur-uracil. Recurrence was observed in 89 patients. A total of 95 patients received gemcitabine as adjuvant chemotherapy or to treat recurrence. The tumor stages were classified according to the Seventh Edition of the Tumor-Node-Metastasis Classification of the Union for International Cancer Center and the Sixth General Rules for the Study of Pancreatic Cancer of Japan Pancreas Society. All clinical samples and patient data were analyzed in accordance with our institutional guidelines and the Declaration of Helsinki after obtaining written informed consent from all patients.

### Immunohistochemical staining and evaluation

The resected surgical specimens were fixed with 10% formaldehyde and embedded in paraffin blocks. The blocks were cut into 2-μm thick sections and mounted on glass slides. The staining protocol was carried out by standard methods, as described previously [34]. The sections were incubated overnight at 4°C with rabbit anti-FBXW7 antibodies (ab109617; dilution, 1:300; Abcam, Cambridge, MA, USA), rabbit anti-MCL1 monoclonal antibodies (ab32087, 1:1000; Abcam), or mouse anti-Ki-67 antibodies (M7240; dilution, 1:150; Dako; Agilent Technologies, Santa Clara, CA, USA) as primary antibodies. Each section was counterstained with Mayer's hematoxylin solution and mounted. A negative control was established by replacing the primary antibody with phosphate-buffered saline (PBS) in 0.1% bovine serum albumin, and no detectable staining was observed.

Assessment of FBXW7 expression was performed as described previously [13]. The expression of nuclear FBXW7 was scored according to the proportion and intensity of staining in five randomly selected fields. We evaluated nuclear FBXW7 expression in the central portion of the tumor, because FBXW7 expression at the cancer invasion front tended to be lower than that at the center of the tumor. The scores for the proportion of stained tumor cell area were as follows: 1, < 10%; 2, 10–50%; 3, > 50%. The scores for the intensity of nuclear staining were as follows: 1, weak; 2, moderate; 3, strong. The final score used in analysis was defined by multiplying the proportion score and intensity score, with a maximum score of 9. We defined the optimal cut-off point as follows: a final score of less than 4 was considered low expression, whereas a score of 4 or more was considered high expression. MCL1 expression was evaluated as low (< 25%) or high (≥ 25%) according to the proportion of positive tumor cells [35]. The expression of Ki-67 was evaluated by calculating the positive cells in 1000 tumor cells for each sample [36]. All images were obtained from a fluorescence microscope (BZ-X700; KEYENCE, Osaka, Japan).

### Cell culture

The human pancreatic cancer cell line SUIT-2 was used in the present study. SUIT-2 cells were obtained from JCRB Cell Bank (Osaka, Japan). The cells were cultured in Dulbecco's modified Eagle medium (Wako, Osaka, Japan) supplemented with 10% fetal bovine serum (FBS) and 1% penicillin-streptomycin (Thermo Fisher Scientific, Kanagawa, Japan) and maintained at 37°C in a humidified 5% CO2 incubator.

### RNAi of FBXW7

FBXW7-specific siRNA (ON-TARGETplus SMARTpool) and nontargeted control siRNA (ON-TARGETplus Non-targeting Pool) were purchased from Dharmacon GE Healthcare (Buckinghamshire, UK). MCL1-specific siRNA was purchased from Thermo Fisher Scientific (Silencer Select siRNA). SUIT-2 cells were suspended at a density of 1.0 × 10^6^ cells in 100 μL Opti-MEM I Reduced Serum Media (Thermo Fisher Scientific) and then mixed with FBXW7-specific siRNA, MCL1-specific RNA, or nontargeted control siRNA as a negative control. Transfection with siRNA was performed using a CUY21 EDIT II electroporator (BEX, Tokyo, Japan), with poring and transfer pulses applied at 150 and 10 V, respectively. Subsequent assays were conducted between 24 and 96 h after transfection.

### Protein extraction and Western blotting

Total protein was extracted from transfected SUIT-2 cells using RIPA Buffer (Wako) according to the manufacturer's protocol. Extracted proteins were separated by sodium dodecyl sulfate polyacrylamide gel electrophoresis with 10% TGX gels (Bio-Rad, Hercules, CA, USA) and transferred to nitrocellulose membranes by the wet transfer method. The membranes were blocked with 5% skim milk and then incubated at 4°C overnight with anti-FBXW7 rabbit monoclonal antibodies (ab171961; 1:1000; Abcam) or anti-MCL1 rabbit monoclonal antibodies (ab32087; 1:1000; Abcam); anti-β-actin mouse monoclonal antibodies (A5316; 1:1000; Sigma, St. Louis, MO, USA) were used as an internal control. The membranes were then treated with horseradish peroxidase-conjugated secondary antibodies. Protein bands on the membrane were detected using ECL Prime Western Blotting Detection Reagent and an Image Quant LAS4000 (GE Healthcare).

### Cell proliferation assays

The proliferation ability of siRNA-transfected SUIT-2 cells was analyzed using a Cell Counting Kit-8 (CCK-8; Dojindo Laboratories, Kumamoto, Japan). SUIT-2 cells were seeded at a density of 2000 cells/well in 96-well plates with 100 μL medium containing FBS. Cell proliferation was evaluated at initial seeding (0 h) and at 24, 48, and 72 h. The reagent was added at a volume of 10 μL/well, and cells were incubated at 37°C for 2 h. The absorbance of each well was measured using an Absorbance Spectrophotometer (Bio-Rad) at 450 nm with the reference wavelength set at 650 nm.

### Wound healing assay

Cell migration ability was evaluated in siRNA-transfected SUIT-2 cells using wound healing assays. The cells were seeded in 24-well plates and cultured until reaching confluence. Then, a uniform straight wound was generated in each using a pipette tip. Each well was washed with PBS to remove all cell debris, and the cells were cultured at 37°C for 48 h. Closure or filling in of the wound was evaluated.

### Invasion assay

Cell invasion assay was performed using 24-well Corning BioCoat Matrigel Invasion Chambers (Corning, NY, USA). Transfected SUIT-2 cells (1 × 10^5^ cells) with 500 μL of medium were seeded in the upper chamber, and the lower chamber was filled with 750 μL of medium containing 10% FBS as a chemoattractant. After a 48 h incubation, the cells were fixed and stained with Diff-Quik (Sysmex Corporation, Kobe, Japan). After staining, the cells which invaded through the pores to the lower surface of the membrane were counted by microscopy. A total of 10 randomly selected fields were evaluated.

### Drug sensitivity assay

Gemcitabine (Selleck Chemicals, Houston, TX, USA) and nab-paclitaxel (Taiho Pharmaceutical, Tokyo, Japan) sensitivities were evaluated using siRNA-transfected SUIT-2 cells. The cells were seeded at a density of 1 × 10^4^ cells/well in 96-well plates with 100 μL medium. After 24 h, the cells were treated with various concentrations of gemcitabine (0, 1, 5, 50, or 100 nM) and nab-paclitaxel (0, 0.1, 1, 5, or 10 μM) for 48 h. Cell viability was evaluated using CCK-8 assays (Dojindo Laboratories), as described above. The absorbance of each well was measured using an Absorbance Spectrophotometer at 450 nm with the reference wavelength set at 650 nm.

### Statistical analysis

Data for continuous variables were expressed as median and range. Statistical significance was analyzed using Mann-Whitney *U* tests for continuous variables and chi-squared tests for categorical variables. Survival curves were described using the Kaplan-Meier method, and differences in survival between groups were compared using the log-rank test. Univariate and multivariate analyses were performed using Cox proportional hazards models and were used to analyze the prognostic factors. Results with *P* values of less than 0.05 were considered statistically significant. All statistical analyses were conducted using the JMP software package (version 13.0.0; SAS Institute Inc., Cary, NC, USA).

## SUPPLEMENTARY MATERIALS FIGURES AND TABLES



## Abbreviations

FBXW7: F-box and WD repeat domain-containing 7
EZH2: enhancer of zeste homolog 2
CCK-8: Cell Counting Kit-8
PBS: phosphate-buffered saline
FBS: fetal bovine serum
RNAi: RNA interference
