# Supplementary material for: Reduced FBXW7 expression in pancreatic cancer correlates with poor prognosis and chemotherapeutic resistance via accumulation of MCL1
Source: Oncotarget. 2017 Nov 6;8(68):112636–46. doi: 10.18632/oncotarget.22634 (PMC5762537; doi:10.18632/oncotarget.22634)
Supplement: Supplementary file 1 [file oncotarget-08-112636-s001.pdf]

Reduced FBXW7 expression in pancreatic cancer correlates with poor prognosis and chemotherapeutic resistance via accumulation of MCL1

SUPPLEMENTARY MATERIALS

Supplementary Table 1: Univariate and multivariate analyses of variables related to relapse-free survival, as determined using Cox proportional hazards models

Variables	Univariate analysis			Multivariate analysis		
	HR	95% CI	*p*-value	HR	95% CI	*p*-value
Age (< 70 versus ≥ 70)	1.02	0.67–1.54	0.921	-	-	-
Sex (male versus female)	0.98	0.65–1.48	0.928	-	-	-
Histological type (well versus moderately/poorly)	1.55	0.87–3.03	0.139	-	-	-
T factor (UICC) (T1, 2 versus T3, 4)	2.92	1.31–8.32	0.006*	1.32	0.57–3.85	0.545
Lymph node metastasis (absent versus present)	1.33	0.83–2.25	0.243	-	-	-
Venous invasion (v0,1 versus v2,3)	2.14	1.40–3.30	< 0.001*	2.09	1.28–3.51	0.004*
Lymphatic invasion (l y 0,1 versus l y 2,3)	1.58	1.04–2.42	0.031*	1.08	0.67–1.75	0.753
Perineural invasion (ne 0,1 versus ne 2,3)	1.55	0.97–2.59	0.07	-	-	-
FBXW7 (high versus low)	1.56	1.02–2.39	0.039*	1.55	1.01–2.37	0.047*

Abbreviations: HR, hazard ratio; CI, confidence interval; UICC, Union for International Cancer Center, *$p < 0.05$.

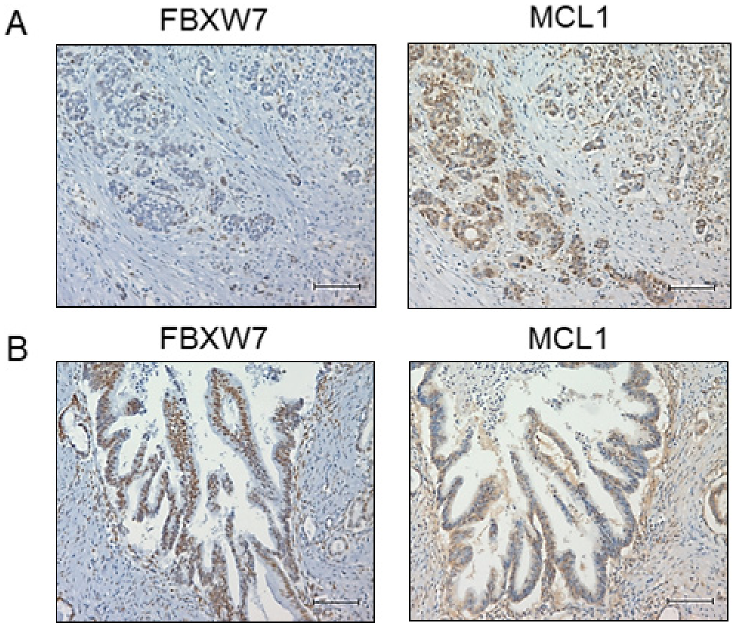

Supplementary Figure 1: Relationship between FBXW7 expression and MCL1 expression, as determined by immunohistochemistry.(**A**) Low FBXW7 expression in tumors was associated with enhanced MCL1 expression. (**B**) High FBXW7 expression in tumors was associated with decreased MCL1 expression. All scar bars are 100 μm.

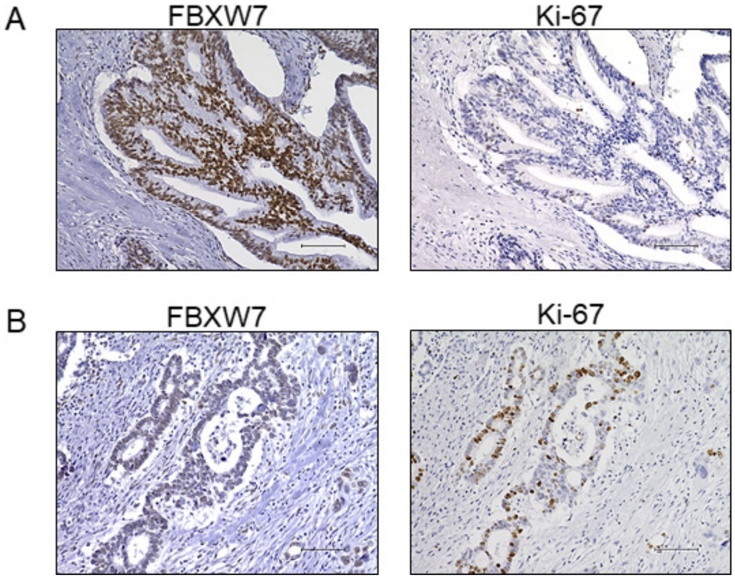

Supplementary Figure 2: Relationship between FBXW7 expression and Ki-67 expression, as determined by immunohistochemistry.Ki-67 expression was inversely correlated with FBXW7 expression. (**A**) High FBXW7 expression was associated with low Ki-67 expression. (**B**) Low FBXW7 expression was associated with high Ki-67 expression. All scar bars are 100 μm.
